# Real-time fusion imaging for guiding transcatheter tricuspid valve repair

**DOI:** 10.1007/s10554-022-02667-z

**Published:** 2022-06-29

**Authors:** Shazia Afzal, Florian Bönner, Tobias Zeus, Malte Kelm, Ralf Westenfeld, Patrick Horn

**Affiliations:** 1 Department of Cardiology, Pulmonology and Vascular Medicine, Medical Faculty of the Heinrich Heine University, Duesseldorf, Germany; 2 Cardiovascular Research Institute, Medical Faculty of the Heinrich-Heine University, Duesseldorf, Germany

## Images in intervention

*Transcatheter tricuspid valve repair (TTVR)* is a promising therapy for severe *tricuspid regurgitation (TR)* that requires accurate navigation within the right heart cavities [1]. We hereby present a novel step-by-step approach to real-time image fusion of transesophageal echocardiography (TEE) and fluoroscopy to simplify device navigation through the right heart for TTVR.

A patient-specific 3D heart model is established based on a 3D full-volume echocardiographic image that is overlaid on fluoroscopy (FI; Anatomical Intelligence, EchoNavigator, Philipps, Best, The Netherlands). The heart model visualizes the echocardiography-derived anatomical cavities, and the fluoroscopy-derived device merged into a single display. In addition, customized anatomic landmarks are set at the anterior and posterior sites of the tricuspid valve in TOE imaging and superimposed on fluoroscopy.

**Fig. 1:**
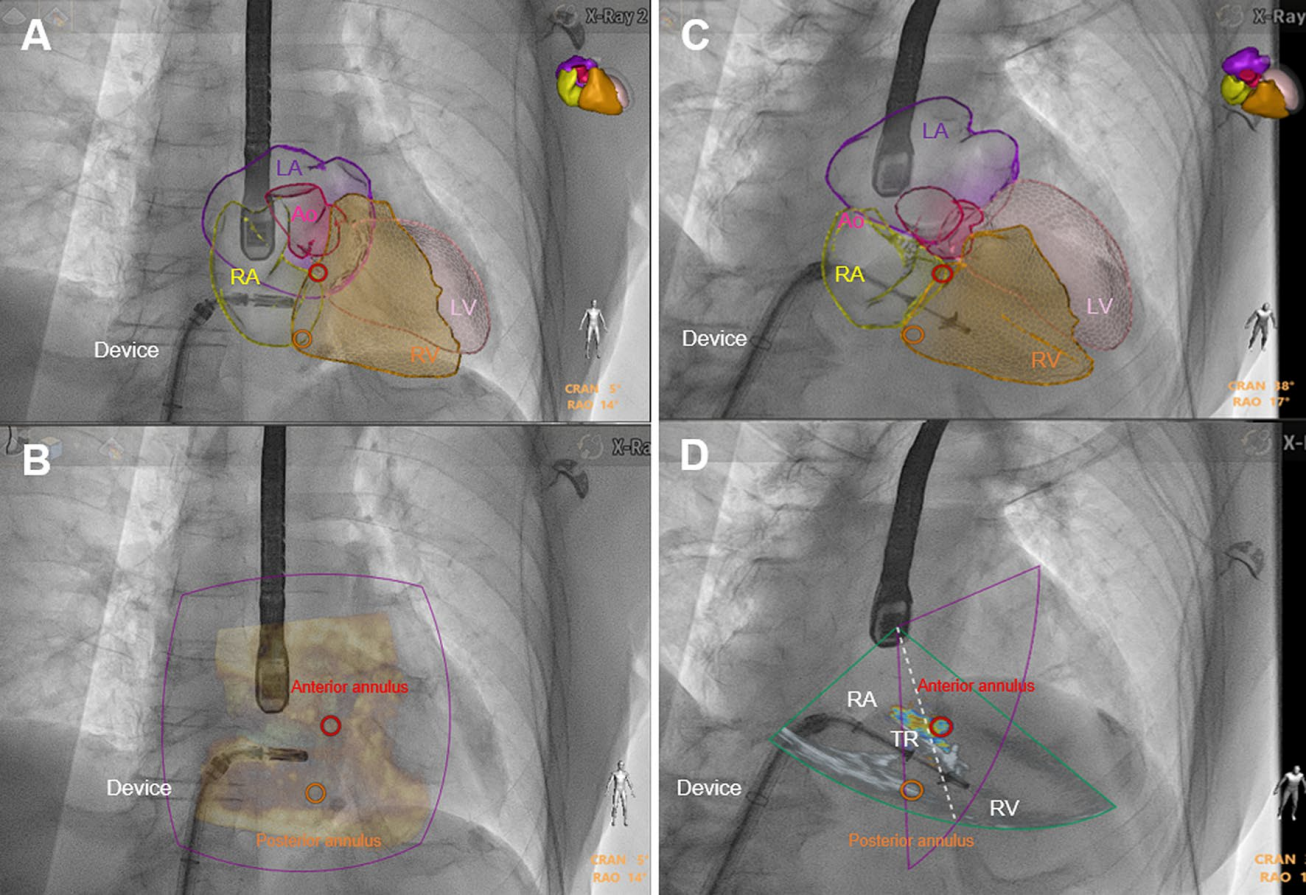
Real-time fusion imaging for guiding TTVR. Fusion of echocardiographic and X-ray images provides the superimposed echo-derived heart cavities and anatomical landmarks (orange marker—posterior anulus, red marker—anterior anulus) onto live X-ray fluoroscopy. **A**, **B** Real-time fusion imaging assisted navigation of the TTVR device through the right atrium pointing to the tricuspid annulus. **C** Safe device passage into the right ventricle. **D** The real-time overlay of the colour doppler onto fluoroscopy helps navigating the clip to valvular regurgitation jets. *RA* right atrium, *LA* left atrium, *RV* right ventricle, *LV* left ventricle, *Ao* aortae, *RVOT* right ventricular outflow tract, *TR* tricuspid regurgitation

The integrated echo-derived right heart cavities on fluoroscopy facilitates device navigation (here the TriClip®, Abbott Vascular GmbH) from the vena cava into the right atrium in the direction of the tricuspid annulus without getting stuck at the interatrial septum or right atrial appendage or causing tissue injury (Fig. 1A, Online Video 1). Real-time 3D-echocardiography overlay visualizes the border of each structure on fluoroscopy and improves the spatial orientation (Fig. 1B). Anatomical details provided by the fusion imaging support efficient manoeuvring of the clip through the valve into the right ventricle (Fig. 1C, Online Video 2). The real-time overlay of the colour doppler onto fluoroscopy helps navigating the clip to valvular regurgitation jets, and the adjustment of the optimal clip position along the commissure (Fig. 1D). The grasping and optimal insertion of tricuspid leaflets on the clip arms are verified by transgastric and deep-oesophageal views before final release (Online Videos 3, 4). Biplane echocardiography image shows successful clip grasping of the tricuspid valve leading to marked TR reduction (Online Video 5).

Here we demonstrate how to use *real-time fusion imaging to simplify TTVR*. Whether fusion imaging guided TTVR improves safety and reduces the procedure time and radiation exposure requires investigation in future trials.

### Supplementary Information

Below is the link to the electronic supplementary material.

Supplementary file1 (MP4 1993 KB) Video 1: Transesophageal 3D-echocardiographic images of the right heart cavities (left panel). Real-time fusion imaging assisted navigation of the TTVR device through the right atrium pointing to the tricuspid annulus (right panel)

Supplementary file2 (MP4 1905 KB) Video 2: Biplane tranesophageal echocardiographic images after safe device passage into the right ventricle (left panel). Fusion of echocardiographic and X-ray images provides the superimposed echo-derived heart cavities and anatomical landmarks (orange marker—posterior anulus, red marker—anterior anulus) onto live X-ray fluoroscopy (right panel)

Supplementary file3 (MP4 1119 KB) Video 3: Biplane transesophageal echocardiographic images showing successful grasping of the septal and posterior leaflet of the tricuspid valve

Supplementary file4 (MP4 1023 KB) Video 4: Transgastric echocardiographic image showing successful grasping of the septal and posterior leaflet of the tricuspid valve

Supplementary file5 (MP4 1458 KB) Video 5: Biplane transesophageal echocardiographic images after leaflet grasping (left panel). Fusion of echocardiographic and X-ray images provides the superimposed echo-derived heart cavities and anatomical landmarks (orange marker—posterior anulus, red marker—anterior anulus) onto live X-ray fluoroscopy (right panel)

## References

[1] Lurz P, Stephan von Bardeleben R, Weber M (2021). Transcatheter edge-to-edge repair for treatment of tricuspid regurgitation. J Am Coll Cardiol.

